# Sex- and age-related differences in arterial pressure and albuminuria in mice

**DOI:** 10.1186/s13293-016-0110-x

**Published:** 2016-11-14

**Authors:** Giannie Barsha, Kate M. Denton, Katrina M. Mirabito Colafella

**Affiliations:** 1 Cardiovascular Program, Monash Biomedicine Discovery Institute, Clayton, Australia; 2 Department of Physiology, Monash University, 26 Innovation Walk (Building 13F), Clayton, VIC 3800 Australia

**Keywords:** Sex, Aging, Menopause, Arterial pressure, Hypertension, Renal function

## Abstract

**Background:**

Animal models have become valuable experimental tools for understanding the pathophysiology and therapeutic interventions in cardiovascular disease. Yet to date, few studies document the age- and sex-related differences in arterial pressure, circadian rhythm, and renal function in normotensive mice under basal conditions, across the life span. We hypothesized that mice display similar sex- and age-related differences in arterial pressure and renal function to humans.

**Methods:**

Mean arterial pressure (MAP) and circadian rhythm of arterial pressure were measured over 3 days via radiotelemetry, in 3- and 5-month-old (adult) and 14- and 18-month-old (aged) FVB/N and in 5-month-old (adult) C57BL/6 male and female normotensive mice. In FVB/N mice, albuminuria from 24-h urine samples as well as body, heart, and kidney weights were measured at each age.

**Results:**

Twenty-four-hour MAP was greater in males than females at 3, 5, and 14 months of age. A similar sex difference in arterial pressure was observed in C57BL/6 mice at 5 months of age. In FVB/N mice, 24-h MAP increased with age, with females displaying a greater increase between 3 and 18 months of age than males, such that MAP was no longer different between the sexes at 18 months of age. A circadian pattern was observed in arterial pressure, heart rate, and locomotor activity, with values for each greater during the active (night/dark) than the inactive (day/light) period. The night-day dip in MAP was greater in males and increased with age in both sexes. Albuminuria was greater in males than females, increased with age in both sexes, and rose to a greater level in males than females at 18 months of age.

**Conclusions:**

Arterial pressure and albuminuria increase in an age- and sex-specific manner in mice, similar to patterns observed in humans. Thus, mice represent a useful model for studying age and sex differences in the regulation of arterial pressure and renal disease. Understanding the mechanisms that underlie the pathophysiology of cardiovascular disease may lead to new and better-tailored therapies for men and women.

## Background

It is well established that age- and sex-related differences exist in the regulation of arterial pressure. During their reproductive years, women have lower average systolic arterial pressure (SAP) and diastolic arterial pressure (DAP) than age-matched men [1–3]. Despite aging being characterized by increases in arterial pressure in both sexes, arterial pressure is generally greater in men than age-matched women. However, the age-related increase in arterial pressure is augmented in 50–60-year-old women such that arterial pressure, particularly SAP, approaches and eventually surpasses that in men [4, 5]. The prevalence and severity of hypertension is therefore greater in postmenopausal women, relative to both premenopausal women and age-matched men [6–10], thereby emphasizing a role for sex differences in the pathophysiology of cardiovascular and renal diseases. Understanding the mechanisms underpinning sex- and age-related differences in the regulation of arterial pressure may uncover new therapies as well as better-tailored treatment of cardiovascular disease (CVD) in both men and women.

Epidemiological studies using 24-h ambulatory blood pressure monitoring (AMBP) in humans have consistently revealed temporal changes in arterial pressure with age [11, 12]. DAP rises until ~50 years of age and subsequently declines, whereas SAP rises from adolescence until old age, suggesting a different significance of DAP and SAP with aging. The Framingham Heart Study was the first to document a relationship between SAP and coronary heart disease risk with advancing age [13]. In healthy humans, arterial pressure exhibits a circadian rhythm, consisting of a nocturnal dip, and a rise in the morning hours, that reaches a peak during the day-time. Accordingly, it is established that SAP and DAP are highest during the day-time and decrease by approximately 10–20% during the nighttime, with day-night differences in arterial pressure being similar in males and females [12, 14, 15]. As arterial pressure increases with age, disturbances in circadian rhythm arise, including diminished nocturnal dipping [16]. Reduced dipping, or even non-dipping of arterial pressure at night, can be more harmful to cardiovascular health than daytime hypertension and is associated with increased risk of end-organ damage and CVD [17–20]. In fact, evidence demonstrates that postmenopausal women are more susceptible to reduced dipping patterns than age-matched men and premenopausal women, due to the augmented increase in arterial pressure they experience with age [17, 21]. In addition, it has been postulated that changes in estrogen levels accompanying menopause may have an effect on dipping status [22]. Preclinical studies in animal models are a mainstay for investigating disease pathophysiology and drug development. It is imperative that strong similarities exist between humans and these animal models if knowledge translation is to be successful.

Similar to humans, other mammalian species also exhibit sexual dimorphism in arterial pressure. Lower arterial pressure in adult females has been well documented in normotensive dogs, sheep, rabbits, rats, and mice as compared to adult males [23, 24]. Furthermore, in rodent, rabbit, and sheep models, it has been shown that females of reproductive age are protected against the development of hypertension, such that arterial pressure increases significantly less in females than in males [25–29]. These findings in different models of hypertension highlight that adult animal models provide an adequate model of the sexual dimorphism in cardiovascular function that exists in humans. However, age-related increases in arterial pressure in animal models have been less well reported. It has been demonstrated that arterial pressure increases with age in sheep [23], rabbits [23, 30], rats [31], and mice [32, 33]. However, these reports often only examined males at different ages, only compare males to females at one age, or at most, males and females at two time points (adult vs aged). Nonetheless, CVD occurs principally in older age groups, and animal studies should include aged cohorts.

Data comparing sex differences in arterial pressure across the lifespan in laboratory animals is limited, and information regarding differences in SAP, DAP, and circadian rhythm is almost non-existent. In the present study, we hypothesized that mice display similar sex- and age-related differences in arterial pressure and renal function to humans and that mice represent a useful model to begin studying sex differences in hypertension. Our aim was to investigate 24-h resting arterial pressures, heart rate (HR), and locomotor activity in conscious male and female normotensive FVB/N mice at 3, 5, 14, and 18 months of age and C57BL/6 mice at 5 months of age via radiotelemetry. In addition, we documented age- and sex-related differences in albuminuria as a marker of renal health.

## Methods

Experiments were conducted in accordance with the Australian Code of Practice for the Care and Use of Animals for Scientific Purposes and approved by the Monash University School of Biomedical Sciences Animal Ethics Committee. Male and female FVB/N and C56BL/6 mice were obtained at 8 weeks of age (Monash Animal Services). Animals were housed in an experimental room with temperature maintained at 24–26 °C and a 12-h light-dark cycle (6 am to 6 pm). All mice were strictly maintained under the same diet, having *ad libitum* access to normal-salt diet (0.26% (wt/wt) NaCl; AIN93M Specialty Feeds, Australia) and water.

### Animal model

Ten groups of virgin mice were studied in total (*n* = 8–12/group). FVB/N male and female mice at four different ages: 3- and 5-month old (adult) and 14- and 18-month old (aged). In addition, C57BL/6 male and female mice were studied at 5 months of age. Serial vaginal smears over 5 days confirmed that all the 14- and 18-month-old female mice were in persistent estrus prior to the commencement of the study. The experimental room was only entered between the hours of 09:00–11:00 or 16:30–17:00 each day.

### Experimental protocol

Two weeks prior to reaching the target age, mice were anesthetized (2.2–2.6% isoflurane in 40% O_2_-60% N_2_, Rhodia, Australia) for implantation of a radiotelemetry probe (TA11PA-C10, Data Sciences International, MN, USA) into the left carotid artery, as described previously [34]. Following a 10-day recovery period, SAP, DAP, mean arterial pressure (MAP), pulse pressure, HR, and locomotor activity were measured by sampling for 10 s every 10 min using Dataquest ART data acquisition system (Data Sciences International, MN, USA) over a 3-day period. Each 24-h period was then overlaid to produce a single 24-h time series and from this data hourly, 12-h (day/night) and 24-h values were calculated. Thereafter, mice were placed in metabolic cages to collect a 24-h urine sample as described previously [35], and albuminuria was measured (Albuwell M mouse albumin ELISA, Exocell Inc, Philadelphia, USA). At the conclusion of the study, animals were anesthetized and heart and kidney weights recorded.

### Statistical analyses

Data are presented as mean ± SEM. Data were analyzed using a two-way ANOVA with the factors sex (*P*_sex_), age (*P*_age_), and their interaction (*P*_sex*age_). This excludes hourly and 12-h arterial pressures, HR, locomotor activity data, which were analyzed using repeated-measures analysis of variance (ANOVA) with the factors sex (*P*_sex_), time (*P*_time_), and their interaction (*P*_sex*time_). Tukey’s post hoc tests were performed where appropriate. *P* ≤ 0.05 was accepted as statistically significant. In addition, for each mouse at each age, the 24-h time series data were further analyzed using the least-squares cosine-fit method to detect 24-h periodicity (GraphPad Prism; version 6.00; GraphPad Software, CA, USA). This cosinor method provided estimates of mesor (middle value of the fitted cosine curve representing the rhythm adjusted mean), amplitude (difference between maximum and mesor of the fitted cosine function), and acrophase (time of peak value of the fitted cosine function). For this analysis, the 9–11 am data, the period when the room was entered for husbandry purposes, was omitted.

## Results

### Sex differences in 24-h arterial pressures, HR, and locomotor activity across the lifespan

**Fig. 1:**
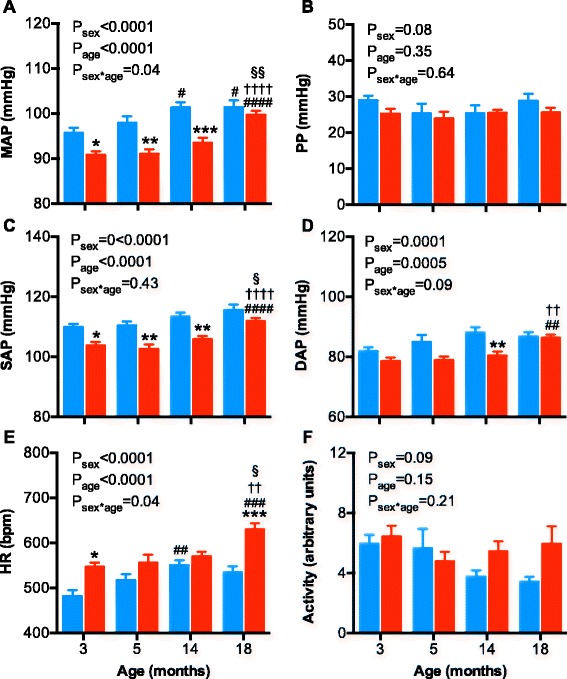
Arterial pressures, pulse pressure, heart rate, and locomotor activity in male and female mice at 3, 5, 14, and 18 months of age. **a** Mean arterial pressure (*MAP*), **b** pulse pressure (*PP*), **c** systolic arterial pressure (*SAP*), **d** diastolic arterial pressure (*DAP*), **e** heart rate (*HR*), and **f** locomotor activity in male (*blue bars*) and female (*red bars*) mice. Data were analyzed using a two-way ANOVA with the factors sex, age, and their interaction. Tukey’s post hoc tests were performed where appropriate. *P < 0.05, **P < 0.01 as compared to age-matched male. ^#^ *P* < 0.05, ^##^ *P* < 0.01, ^###^ *P* < 0.001 as compared to 3-month-old counterpart. ^†^ *P* < 0.05, ^†††^ *P* < 0.001 as compared to 5-month-old counterpart. ^§^ *P* < 0.05 as compared to 14-month-old counterpart

Twenty-four-hour MAP was greater in males than females (*F*_(1, 75)_ = 42.6, *P*_sex_ < 0.0001), being ~5 mmHg (*P* < 0.05), ~7 mmHg (*P* < 0.005) and ~8 mmHg (*P* < 0.0001) greater in males than females at 3, 5, and 14 months of age, respectively (Fig. 1a). At 18 months of age, 24-h MAP was not significantly different between male and female mice (~2 mmHg; *P* = 0.9). Twenty-four-hour MAP increased with age in both sexes (*F*_(3, 75)_ = 16.0, *P*_age_ < 0.0001). In males, arterial pressure was not different between 3 and 5 months but had increased significantly by ~6 mmHg at 14 months of age (*P* < 0.05), at which time MAP plateaued as no further increase was observed by 18 months of age (Fig. 1a). Contrastingly, MAP in females did not increase significantly until 18 months of age (~9 mmHg compared to 3 months of age, *P* < 0.0001), when 24-h MAP was no longer significantly different to that observed in males (Fig. 1a). Thus, the increase in 24-h MAP between 3 and 18 months of age was significantly greater in females than males (*F*_(3, 75)_ = 2.7, *P*_age*sex_ < 0.05). However, pulse pressure was not different between the sexes at any age (Fig. 1b), as similar age- and sex-related changes were observed in SAP (Fig. 1c) and DAP (Fig. 1d).

Average 24-h HR was sex-dependent (*F*_(3, 75)_ = 32.1, *P*_sex_ < 0.0001) and increased with age (*F*_(3, 75)_ = 9.8, *P*_age_ < 0.0001), but this increase was greater in females than males (*F*_(3, 75)_ = 3.0, *P*_sex*age_ < 0.04; Fig. 1e). Twenty-four-hour HR was greater in females than males at 3 months of age, being ~65 bpm greater (~14%, *P* < 0.05; Fig. 1e). Twenty-four-hour HR was not significantly different between the sexes at 5 and 14 months of age, principally due to an increase in 24-h HR in males. At 14 months of age, 24-h HR increased by ~70 bpm (~15%; *P* < 0.01) as compared to 3 months of age in males. In contrast, 24-h HR was similar between the females at 3, 5, and 14 months of age but rose by ~84 bpm (~15%, *P* < 0.0001) at 18 months compared to 3 months of age in females. Thus, at 18 months of age, 24-h HR was ~96 bpm (~18%, *P* < 0.0001) greater in female than age-matched male mice (Fig. 1e).

Locomotor activity was not significantly different between the sexes (Fig. 1f; *F*_(3, 75)_ = 2.8, *P*_sex_ = 0.09) nor did locomotor activity decrease significantly with age (Fig. 1f; *F*_(3, 75)_ = 1.8, *P*_age_ = 0.15). However, locomotor activity was highly variable. To increase statistical power, in post hoc analysis, the young (3- and 5-month old) and aged (14- and 18-month old) males and females were pooled. In the young mice (3–5 months of age), locomotor activity was similar in the male (5.8 ± 0.6 arbitrary units, *n* = 18) and female mice (5.6 ± 0.5 arbitrary units, *n* = 23). In the aged mice (14–18 months of age), locomotor activity declined in male (3.5 ± 0.3 arbitrary units, *n* = 18, *P* = 0.05) but not in female mice (5.7 ± 0.6 arbitrary units, *n* = 23, *P* = 0.9).

**Fig. 2:**
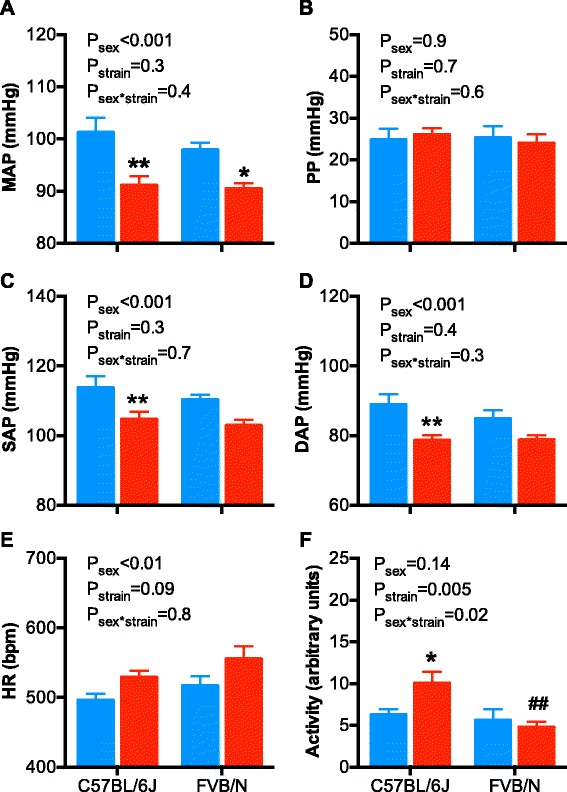
Arterial pressures, pulse pressure, heart rate, and locomotor activity in male and female C57BL/6 and FVB/N mice at 5 months of age. **a** Mean arterial pressure (*MAP*), **b** pulse pressure (*PP*), **c**) systolic arterial pressure (*SAP*), **d** diastolic arterial pressure (*DAP*), **e** heart rate (*HR*), and **f** locomotor activity in male (*blue bars*) and female (*red bars*) mice. Data were analyzed using a two-way ANOVA with the factors sex, strain, and their interaction. Bonferroni’s post hoc tests were performed where appropriate. *P < 0.05, **P < 0.01 as compared to the male of the same strain. ^##^ *P* < 0.01 as compared to the C57BL/6 female

At 5 months of age, arterial pressures, HR, and locomotor activity were measured in C57BL/6 mice (Fig. 2a–f). In this strain, MAP was greater in male than in female mice (~10 mmHg), which was similar to that observed in the FVB/N stain (Fig. 2a; *P*_sex_ < 0.0001, *P*_strain_ = 0.4, *P*_sex*strain_ = 0.3). A similar pattern was observed for SAP and DAP, with pulse pressure not different between the sexes or strains (Fig. 2b–d). HR was greater in the C57BL/6 females than in males, similar to the FVB/N strain (Fig. 2e, *P*_sex_ < 0.01). The most notable difference between the strains was the greater locomotor activity in the C57BL/6 female mice which was significantly greater than males of the same strain and males and females of the FVB/N strain (Fig. 2f, all *P* < 0.05).

### Sex- and age-related changes in the circadian pattern of arterial pressure, HR, and locomotor activity

**Fig. 3:**
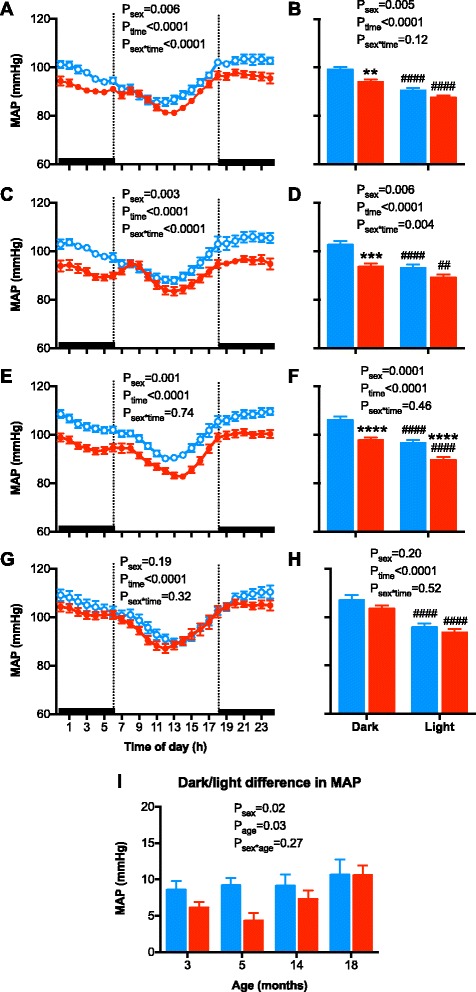
Hourly mean arterial pressure (MAP) and 12-h MAP of the dark and light periods of the day in **a**, **b** 3-month-old; **c**, **d** 5-month-old; **e**, **f** 14-month-old; and **g**, **h** 18-month-old male (*blue symbols/bars*) and female (*red symbols/bars*) mice. **i** The change in MAP from the dark to light period of the day in male (*blue bars*) and female (*red bars*) mice at 3, 5, 14, and 18 months of age. Shading indicates the 12-h dark/light cycle (lights off at 18:00). Hourly data were analyzed using repeated measured ANOVA with the factors sex, time, and their interaction. Twelve hours data were analyzed using an ANOVA with the factors sex, time, and their interaction followed by Bonferroni’s post hoc tests. The dark/light change in MAP data was analyzed using a two-way ANOVA with the factors sex, age, and their interaction followed by a Tukey’s post hoc tests. ^##^ *P* < 0.01, ^####^ *P* < 0.0001 as compared to respective dark period. **P < 0.01, ***P < 0.001, ****P < 0.0001 as compared to respective period in male mice

**Fig. 4:**
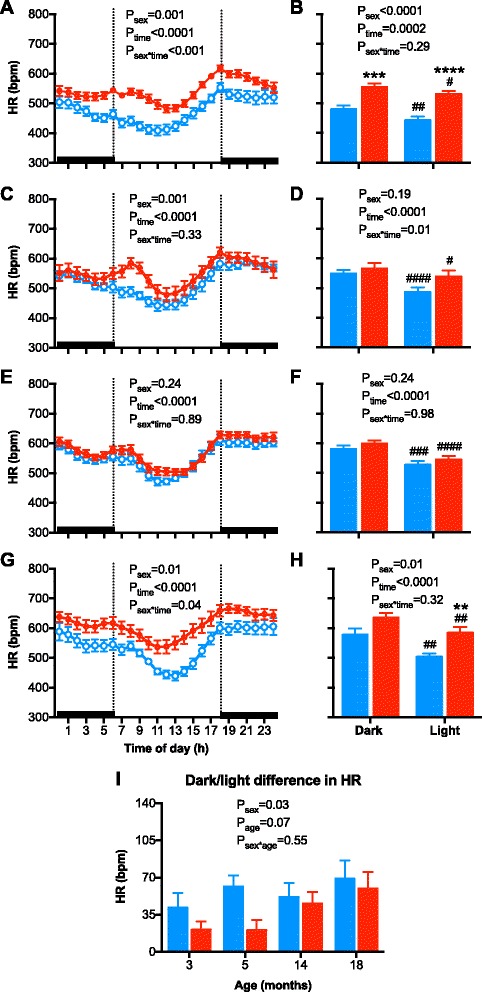
Hourly heart rate (HR) and 12-h HR of the dark and light periods of the day in **a**, **b** 3-month-old; **c**, **d** 5-month-old; **e**, **f** 14-month-old, **g**, **h** 18-month-old male (*blue symbols/bars*) and female (*red symbols/bars*) mice. **i** The change in HR from the dark to light period of the day in male (*blue bars*) and female (*red bars*) mice at 3, 5, 14, and 18 months of age. *Shading* indicates the 12-h dark/light cycle (lights off at 18:00). Hourly data were analyzed using repeated measured ANOVA with the factors sex, time, and their interaction. Twelve hours data were analyzed using a two-way ANOVA with the factors sex, time, and their interaction followed by Bonferroni’s post hoc tests. The dark/light change in HR data were analyzed using a two-way ANOVA with the factors sex, age, and their interaction followed by a Tukey’s post hoc tests. ^#^ *P* < 0.05, ^##^ *P* < 0.01, ^###^ *P* < 0.001, ^####^ *P* < 0.0001 as compared to respective dark period. **P < 0.01, ***P < 0.001, ****P < 0.0001 as compared to respective period in male mice

**Fig. 5:**
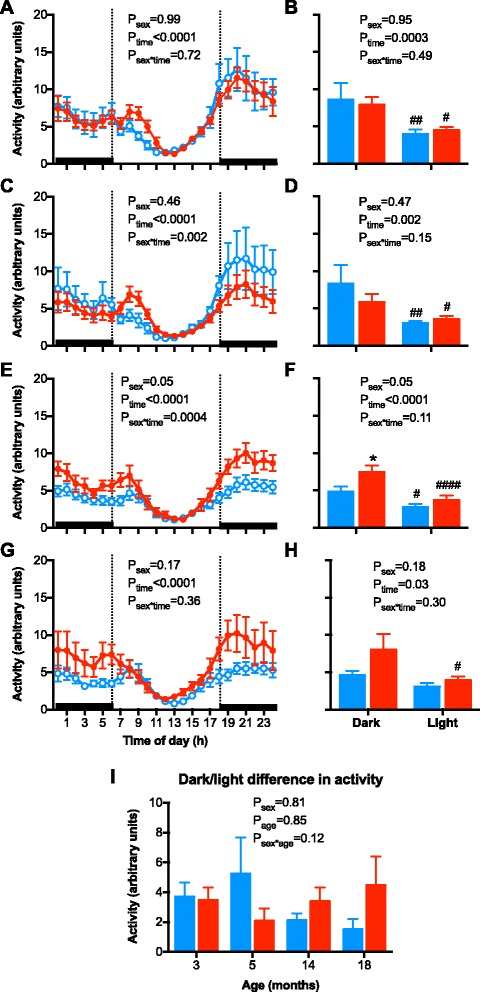
Hourly locomotor activity (activity) and 12-h activity of the dark and light periods of the day in **a**, **b** 3-month-old; **c**, **d** 5-month-old; **e**, **f** 14-month-old; and **g**, **h** 18-month-old male (*blue symbols/bars*) and female (*red symbols/bars*) mice. **i** The change in HR from the dark to light period of the day in male (*blue bars*) and female (*red bars*) mice at 3, 5, 14, and 18 months of age. *Shading* indicates the 12-h dark/light cycle (lights off at 18:00). Hourly data were analyzed using repeated measured ANOVA with the factors sex, time, and their interaction. Twelve hours data were analyzed using a two-way ANOVA with the factors sex, time, and their interaction followed by Bonferroni’s post hoc tests. The dark/light change in activity data were analyzed using a two-way ANOVA with the factors sex, age, and their interaction followed by a Tukey’s post hoc tests. *P < 0.05 as compared to respective period in male mice. ^#^ *P* < 0.05, ^##^ *P* < 0.01, ^####^ *P* < 0.0001 as compared to respective dark period

A circadian pattern was observed in arterial pressure, HR, and locomotor activity (Figs. 3, 4, and 5; all *P*_time_ < 0.0001) with values for each being greater during the dark period (18:00 to 06:00 h; night/active period) as compared to the light period (06:00 to 18:00 h; day/inactive period). A spike in measures was observed between 09:00 to 11:00 h in the morning. During this time, the room was entered by staff for experimental and husbandry purposes.

MAP measured each hour over a 24-h period was greater in male than female mice at 3, 5, and 14 months of age (Fig. 3a, c, e; all *P*_sex_ < 0.01), similar to the averaged 24-h MAP data (Fig. 1a). Whereas, at 18 months of age, hourly MAP was no longer significantly different between the sexes (Fig. 3g; *F*_(1, 18)_ = 1.8, *P*_sex_ = 0.2). Similarly, 12-h light and dark period MAP was higher in males than in females at 3, 5, and 14 months of age (Fig. 3b, d), but not at 18 months of age (Fig. 3h). Calculation of dark-light difference in MAP (Fig. 3i) demonstrated an age- and sex-related difference in the dip in MAP (~5–10 mmHg), but an interaction between sex and age was not detected (*F*_(3, 75)_ = 3.1, *P*_age_ = 0.03; *F*_(1, 75)_ = 6.1, *P*_sex_ = 0.02; *F*_(3, 75)_ = 1.0, *P*_sex*age_ = 0.3).

Hourly HR over a 24-h period, as observed in the averaged 24-h HR data (Fig. 1e), was significantly greater in female at 3 and 18 months of age (Fig. 4a, g, both *P*_sex_ < 0.01), but not at 5 or 14 months of age (Fig. 4c, e). The greater HR in females at 3 and 18 months of age occurred in both the light and dark periods (Fig. 4b, h). No significant difference was observed in the dark-light dip in HR between the sexes at any age (Fig. 4i).

Hourly locomotor activity over a 24-h period was not significantly different between the sexes at 3 and 5 months of age (Fig. 5a–d), as observed in the averaged 24-h locomotor data (Fig. 1f). Locomotor activity dipped by a similar extent in males and females between the light and dark periods at 3 and 5 months of age (Fig. 5b, d, i). The greater 24-h locomotor activity observed in the aged females (pooled 14 and 18 months data) as compared to the males (Fig. 1f) was due to an increase in locomotor activity during the dark and not the light period (Fig. 5e–h).

In addition to the analysis of the diurnal rhythm of MAP, HR, and locomotor activity described above, we also fitted the data using cosinor analysis. This data is represented in Table 1. Using this method of analysis, a similar pattern of change in MAP, HR, and locomotor activity with age and between the sexes was observed, as seen with the more simple method described above (24-h, 12-h, and 1-h averages). Examination of the peak amplitude did not detect significant differences across age or sex in MAP, HR, or locomotor activity. It was identified that the peak increase in MAP, HR, and locomotor activity occurred between 21 and 24:00 h and that this pattern was not altered by sex or age.

**Table 1:** Mesor (average), amplitude and acrophase of MAP, heart rate, and locomotor activity in male and female C57BL6 mice at 3, 5, 14, and 18 months (Mo) of age over a 24-h cycle time (CT; midnight to midnight) following cosinar analysis

	M 3-Mo	F 3-Mo	M 5-Mo	F 5-Mo	M 14-Mo	F 14-Mo	M 18-Mo	F 18-Mo
n	12	11	11	7	12	10	12	8
MAP								
Mesor (mmHg)	95 ± 1	90 ± 1*	98 ± 1	91 ± 1*	101 ± 1^#^	93 ± 1**	101 ± 1^#^	98 ± 1^##^
Amplitude (mmHg)	9 ± 1	7 ± 1	9 ± 1	6 ± 1	9 ± 1	8 ± 1	9 ± 1	9 ± 1
Acrophase, CT (h)	22.2 ± 0.8	22.5 ± 0.8	23.5 ± 1.0	22.0 ± 0.8	22.6 ±	21.0 ± 1.8	23.7 ± 0.8	23.4 ± 1.1
HR								
Mesor (bpm)	473 ± 14	539 ± 11*	514 ± 14	546 ± 19	549 ± 11^##^	567 ± 10	535 ± 16^#^	607 ± 17*^##^
Amplitude (bpm)	64 ± 8	48 ± 4	70 ± 7	52 ± 5	63 ± 6	58 ± 6	77 ± 9	52 ± 9
Acrophase, CT (h)	21.3 ± 0.6	21.8 ± 1.6	20.5 ± 0.6	20.7 ± 0.7	21.0 ± 0.9	20.7 ± 0.7	22.4 ± 1.0	20.3 ± 0.7
Activity								
Mesor (units)	4.9 ± 0.3	5.8 ± 0.6	4.4 ± 0.4	4.1 ± 0.6	3.5 ± 0.4	5.3 ± 0.6	3.4 ± 0.4	5.9 ± 1.2
Amplitude (units)	3.3 ± 0.4	3.6 ± 0.6	3.2 ± 0.4	2.3 ± 0.6	2.0 ± 0.4	3.5 ± 0.6	2.0 ± 0.4	3.1 ± 0.7
Acrophase, CT (h)	20.6 ± 1.5	22.3 ± 0.9	21.8 ± 1.0	18.5 ± 2.2	18.5 ± 1.9	21.9 ± 1.2	19.3 ± 2.2	21.5 ± 1.0

*P < 0.05, **P < 0.01 compared to age-matched males; ^#^ *P* < 0.05, ^##^ *P* < 0.01 as compared to 3 months of age in the same sex

### Sex- and age-related differences in body and organ weights

**Fig. 6:**
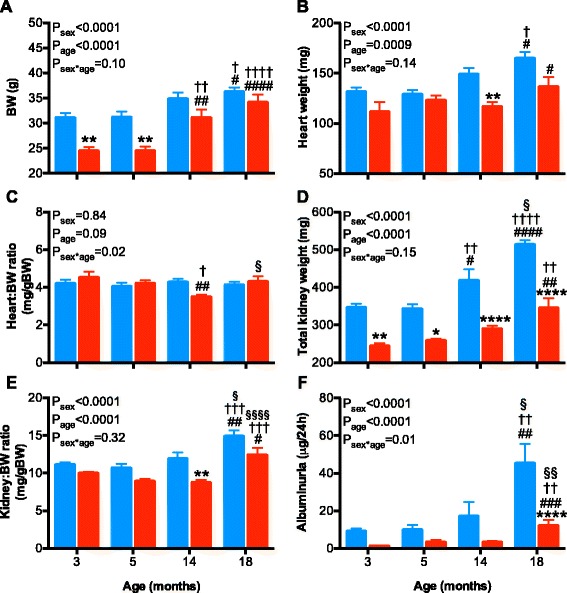
Body weight, organ weights, and albuminuria in male (*blue bars*) and female (*red bars*) mice at 3, 5, 14, and 18 months of age. **a** Body weight (*BW*), **b** heart weight, **c** heart weight to BW ratio, **d** total kidney weight, **e** total kidney weight to BW ratio, and **f** albuminuria. Data were analyzed using a 2-way ANOVA with the factors sex, age and their interaction. Tukey post hoc tests were performed where appropriate. *P < 0.05, **P < 0.01, ****P < 0.0001 as compared to age-matched male. ^#^ *P* < 0.05, ^##^ *P* < 0.01, ^###^ *P* < 0.001, ^####^ *P* < 0.0001 as compared to the 3-month-old counterpart. ^†^ *P* < 0.05, ^††^ *P* < 0.01, ^†††^ *P* < 0.001, ^††††^ *P* < 0.001 as compared to the 5-month-old counterpart. ^§^P < 0.05, ^§§^P < 0.01, ^§§§§^P < 0.0001 as compared to the 14-month-old counterpart

There were no unexplained deaths in any groups throughout the study. Mice were lost from the study at each age group due to surgical losses or telemetry probe failure, but this was similar for each age and sex (~5% per group). Body weight was ~22% greater in adult (3- and 5-month old) male than female mice (both *P* < 0.01, Fig. 6a). Body weight increased with age in both sexes (*F*_(3, 75)_ = 22.71, *P*_age_ < 0.001). In males, body weight was not different between 3 to 14 months of age but was significantly greater by ~16% at 18 months, as compared to 3 months of age (*P* < 0.05). There was no further significant increase by 18 months of age in the males. In females, body weight was not different between 3 to 5 months but was markedly greater by ~30% (*P* < 0.01) at 14 as compared to 3 months of age. There was no further significant increase in body weight between 14 to 18 months of age in the female mice (Fig. 6a).

Heart weight increased with age in both sexes (Fig. 6b, *F*_(3, 59)_ = 6.318, *P*_age_ = 0.0009). Heart weight was significantly greater at 18 months of age as compared to both 3-month - old males and females (Fig. 6b, both *P* < 0.05). Heart weight was not significantly different at 3 and 5 months of age between males and females but was greater in males at 14 months of age (Fig. 6b, *P* < 0.01) and tended to be greater at 18 months of age (Fig. 6b, *P* = 0.16). Heart to body weight ratio was similar between the groups, except at 14 months, when heart to body weight ratio was lower in females as compared to 3-, 5-, and 18-month-old females (all *P* < 0.05). This was primarily due to an age-related increase in body weight in the females at 14 months of age (Fig. 6a).

Total kidney weight was lower in females as compared to males at all ages (Fig. 6d). In males, kidney weight was greater at 14 and 18 months of age as compared to younger males. Conversely, in females, kidney weight was similar at 3, 5, and 14 months of age. However, by 18 months of age, kidney weight was significantly increased in females as compared to younger females. The kidney to body weight ratio increased with age in both sexes (Fig. 6e, *F*_(3, 59)_ = 19.93, *P*_age_ < 0.0001), such that kidney weight was greater in 18-month-old males and females as compared to their 3-, 5-, and 14-month-old counterparts.

### Sex differences in the age-related increase in albuminuria

Albuminuria was assessed via collection of a 24-h urine sample. Albuminuria increased with age (*F*_(1, 50)_ = 12.49, *P*_age_ < 0.0001) and sex (*F*_(1, 50)_ = 23.61, *P*_sex_ < 0.0001), with the increase across age greater in males than females (*F*_(1, 50)_ = 4.05, *P*_sex*age_ = 0.01; Fig. 6f). In both sexes, albuminuria was greater at 18 months as compared to 3, 5, and 14 months of age (Fig. 6f). Between the sexes, post hoc analysis did not detect significant differences in albuminuria at 3, 5, or 14 months of age (Fig. 6f). However, at 18 months of age, when albuminuria was increased by ~4-fold in males and ~7.5-fold in females (as compared to their 3-month-old counterparts, both *P* < 0.01), albuminuria was still significantly lower in females as compared to males (*P* < 0.0001).

## Discussion

The present study demonstrated age- and sex-related differences in arterial pressure and circadian rhythm across the lifespan of normotensive mice. MAP was significantly greater in males than females at 3, 5, and 14 months of age and increased in both sexes with age. However, the increase was steeper in females such that by 18 months, MAP was no longer significantly different between the sexes. A circadian rhythm was established in both sexes for MAP, HR, and locomotor activity, with these variables peaking during the dark, active period and dipping during the light, inactive period. In addition, albuminuria showed a sexually dimorphic pattern, being greater in males and increasing to a greater level with age in males. This study highlights that arterial pressure tracks with age and sex in mice, as in humans, and provides direction with respect to the timing of future studies that aim to investigate sex differences in arterial pressure and renal function. It also provides information about the sample size that may be required, in order to detect differences in MAP between the sexes with confidence.

Arterial pressure was greater in young adult male mice at 3 and 5 months of age as compared to age-matched females. This sex difference in arterial pressure was observed in both FVN/B and C57BL/6 mouse strains at 5 months of age. The age, 3–5 months (12–20 weeks), is the most common range for studies to be conducted in mice. Moreover, the sex difference in resting arterial pressure, in the order of ~5 mmHg, is consistent with previous reports in mice and other mammalian species, including humans (mice [36], rats [31], rabbits [30], sheep [23], dogs [37], and human [12]). However, not all studies have detected a sex difference in arterial pressure. This was the case when Deschepper and colleagues [29] measured SAP (tail-cuff) in 13 strains of mice and discovered sex differences in only four strains; a sex difference in SAP was not detected in the FVB/N strain under baseline conditions contrary to our data. Additionally, a sex difference in SAP or DAP was not identified in 12-week-old rats [38], 3–4-month-old guinea pigs [39], or adult Clawn miniature pigs [40]. Nonetheless, it is important to recognize that failure to detect dimorphism in arterial pressure between the sexes most likely reflects the lack of statistical power and/or the low sensitivity of the method used to measure arterial pressure. Radiotelemetry, as used in the current study, is the gold standard for arterial pressure measurement in rodents, allowing for unrestrained, 24-h recordings in their home environment [41]. In the present study, the sex difference in MAP over a 24-h period was 5 ± 4 mmHg (mean ± SEM) in the 3-month-old mice. Thus, the sample size calculation *N* = 2s^2^C/(relative difference)^2^ (where *s* equals the standard deviation and *C* is a constant assuming a level of significance of 0.05 and power of 0.8 (7.85) and a relative difference of 5 mmHg in blood pressure between the sexes) demonstrates that *n* = 10/group is required to detect this level of difference in MAP between groups with confidence. Studies utilizing the tail-cuff plethysmography to measure arterial pressure in mice report a standard deviation of ~8 mmHg [29, 42, 43] and would therefore, with a sample size of *n* = 10, be able to detect a ~10 mmHg mean difference in arterial pressure between groups. Alternatively, a sample size of *n* > 30 would be required to detect a mean difference of ~5 mmHg between groups. A limitation of the current study was the cross-sectional nature of the data collection. This was unavoidable as the radiotelemetry probes are designed with a battery life of 6 weeks, and as a result, it was not possible to measure MAP from 3–18 months of age in the same mice via radiotelemetry. This compounds the difficulty in detecting age differences in arterial pressure. Consequently, it is not surprising that sex differences in arterial pressure often go undetected.

Whilst studies have not consistently demonstrated sex differences in resting arterial pressure in healthy animals, reports of sexual dimorphism in arterial pressure are more consistent in rodent models of hypertension [24]. Angiotensin II (AngII) infusion is a common experimental model of induced hypertension. Chronic AngII infusion increases arterial pressure to a higher level in adult male mice and rats than in adult females [26–28, 44]. Studies in the adult spontaneously hypertensive rat (SHR) [45, 46], Dahl-sensitive [47, 48], and New Zealand genetically hypertensive [49] rats have also reported higher arterial pressure in males relative to females. Moreover, in SHR, the rise in arterial pressure from 2 to 5 months is more rapid in onset and greater in males than in females [5, 46]. This sexual dimorphism then disappears by 16–18 months of age, such that female arterial pressure becomes similar, or exceeds that of age-matched males, much like the trend we have observed with FVB/N mice [50].

Arterial pressure increased with age in male and female mice, with a steeper rise in females. In line with previous findings, female mice displayed no significant differences in MAP between 3 and 14 months of age [36, 51]. However, a marked increase in arterial pressure occurred at 18 months of age in the females (~9 mmHg). The increase in MAP in the aged females was associated with reproductive senescence. Mice enter persistent estrus, depending upon strain, by 11–16 months of age [52, 53]. In the current study, although 14-month-old female mice were in persistent estrus, arterial pressure did not increase until 18 months of age. This suggests that there is a period following the loss of estrus cyclicity when the protective actions of estrogen wane. In contrast, MAP in males increased between 3 and 14 months of age (~6 mmHg) but did not increase further by 18 months of age. This extends our earlier findings that MAP is significantly higher in aged (14-month old) relative to adult (4-month old) male mice [36, 51]. Conversely, a report of SAP in mice from 3–30 months of age (plethesmography; *n* = 6–8) did not detect a significant increase in SAP between the sexes or age [54]. Contrariwise, a study in rats from 3–16 months of age revealed both sex- and age-related differences in SAP, and linked these changes with the loss of estrus cyclicity and plasma testosterone levels [31]. Similarly, the age-related increase in arterial pressure in 18-month-old reproductively senescent female SHR was associated with a reduction in plasma estrogen and an increase in plasma testosterone levels [50]. A limitation of the current study was that estrogen levels were not determined. Sex chromosomes and hormones during reproductive age are known to differentially regulate the renin-angiotensin system and provide cardiovascular protection and are therefore likely to contribute to sex differences in arterial pressure [55]. Thus, sexual dimorphism in the tracking of arterial pressure with age is associated with factors that lead up to, as well as accompany, the transition to reproductive senescence.

Mice displayed a sexually dimorphic pattern in the increase in arterial pressure with age, which is similar to humans, at least in terms of MAP. In humans, MAP increases throughout adulthood until it reaches a plateau in the seventh decade of life [11, 56]. However, in mice, SAP and DAP increased by a similar extent over time, such that pulse pressure was unaffected by age in either sex. This is in contrast to humans, in which there is a marked increase in pulse pressure around the age of 50, with the increase being substantially greater in females [2, 57, 58]. In terms of HR, clinical studies indicate that normotensive adult women have a higher resting HR than adult men [59] and a similar finding was observed in mice. Thus, with respect to changes in pulse pressure with age, mice did not completely mimic the human condition. Several possible explanations could account for this dichotomy. The mice at 18 months of age may not be old enough to exhibit such changes, or changes in pulse pressure were beyond the limits of detection. Alternatively, the smaller mouse heart and relative size of the vasculature may not respond in the same manner as humans, to the effects of aging. The location of the measurement of arterial pressure (carotid arterial vs forearm) may also underlie the difference. Finally, environmental influences such as physical activity and diet are likely to have a greater impact on arterial pressure in an aging human population than a mouse that is maintained under standardized sedentary laboratory housing conditions. In humans, it has been reported that males are more physically active (accelerometer) than females [60]. We detected an increase in locomotor activity between the sexes in young mice. This discrepancy may reflect the possibility that locomotor activity in mice and accelerometer data in humans may not measure the same components of activity, or may reflect the fact that the mice were housed under sedentary conditions. In addition, mice are a commonly used model of human disease, and it is being increasingly recognized that (patho)-physiological differences exist between strains [29]. Whilst there was no difference in MAP and HR between the FVN/B and C57Bl6 strains at 5 months of age, locomotor activity was considerably greater in female C57BL6 mice, which may affect the age-related increase in MAP. Future studies should take into account possible age-, sex-, and strain-related differences in cardiovascular and renal function.

It is well established that arterial pressure exhibits a circadian variation during each wake-sleep state in mammals, including humans [61] and mice [26, 62]. Accordingly, in mice, we observed a circadian pattern in arterial pressure, HR, and locomotor activity. In mice, as in humans, arterial pressure dips during the period when sleep and rest predominate (12-h day/light period for mice), irrespective of gender. This was associated with decreased HR and locomotor activity. During the period when wakefulness and activity predominate (12-h night/dark period for mice), arterial pressure, HR, and locomotor activity rise. Consistent with the literature, MAP of mice in the night was higher than in the daylight hours [63, 64]. In C57BL/6 mice, the rise in arterial pressure during the night-time active period is understood to be mediated by an increase in cardiac output (CO), which is directly linked to increased HR and locomotor activity [65]. Likewise, in humans, the increase in arterial pressure during wakefulness is related to an increase in CO that is attributed to an increase in HR and physical activity, a decrease in total peripheral resistance, with little to no changes in stroke volume [66]. Taken together, the circadian rhythm in mice is similar to that in humans and is caused by similar hemodynamic changes, which renders the mouse model relevant for studies assessing the management and prevention of CVD.

The dip in MAP (~5 mmHg) during the inactive period was apparent in both sexes in mice. Statistically, a main effect of age and sex was observed, indicating that overall, the dip in MAP was greater in males and increased with age in both sexes. Despite the circadian dip in MAP appearing to be attenuated in adult female mice, no significant difference, at any specific age, was discerned. It is likely that this apparent difference reflected the increased reactivity of adult female mice to environmental stress. The adult female mice certainly had a greater spike in MAP during the hours of 9:00–11:00, when the radiotelemetry laboratory was entered for husbandry and experimental purposes. However, cosinor analysis, in which the data from the period of 9–11 am was omitted, did not detect a difference in wave amplitude. Thus, no marked sex- or age-related difference in the circadian dip in MAP was observed in mice. In contrast, arterial pressure in adult women dips lower at night then in men, a difference that is lost with age [67, 68]. Extension of these studies into disease models may expose perturbations in the circadian rhythm of arterial pressure in mice. A reduction in arterial pressure dipping during sleep is a hallmark of early dysregulation in arterial pressure, with poor prognostic implications for CVD [69, 70].

It is well established in animals and humans that renal function decreases with age, with the decline commencing earlier in males than females [71–73]. In the current study, albuminuria was documented at each age as a marker of renal health. Overall, male mice at all ages excreted a greater amount of albumin each day than female mice. This is in agreement with a study in rats, that reported greater proteinuria in adult males than females [74]. Albuminuria increased markedly with age in mice, in association with increasing MAP and body and kidney weight in both sexes. Evidence suggests that this decline in renal function is due to alterations in the glomerular filtration barrier [75]. Moreover, these changes are correlated to higher testosterone levels in males, and in females, to an increase in the testosterone to estrogen balance following reproductive senescence [76].

## Conclusions

Our study demonstrates that similar to humans, arterial pressure and albuminuria increase in a sex- and age-specific manner in mice, including different strains. We highlight that sex- and age-related differences in arterial pressure can be difficult to identify and requires large sample sizes in order to detect these differences with confidence. Moreover, our study reveals that mice represent a useful model for studying sex- and age-related differences in the pathophysiology of hypertension and renal disease. It is essential in future studies to examine aged mice of both sexes to delineate the underlying mechanisms causing sex- and age-related differences in arterial pressure, as this may lead to the identification of new targets for the treatment of CVD.
